# *N*′-(2,6-Dichloro­benzyl­idene)-2-hy­droxy­benzohydrazide

**DOI:** 10.1107/S1600536810035385

**Published:** 2010-09-25

**Authors:** Yawar Baig, Hamid Latif Siddiqui, Waseeq Ahmad Siddiqui, Ghulam Mustafa, Harald Krautscheid

**Affiliations:** a Institute of Chemistry, University of the Punjab, Lahore, Pakistan; b Department of Chemistry, University of Sargodha, Sargodha, Pakistan; c Institute of Inorganic Chemistry, Leipzig University, Johannisallee 29, D-04103 Leipzig, Germany

## Abstract

In the title compound, C_14_H_10_Cl_2_N_2_O_2_, the dihedral angle between the two aromatic rings is 17.39 (4)°. An intra­molecular O—H⋯O hydrogen bond forms a six-membered *R*(6)_1_^1 ^ring motif. In the crystal structure, inter­molecular N—H⋯O and O—H⋯O hydrogen-bonding inter­actions occur.

## Related literature

For the biological activity of Schiff bases, see: El-Masry *et al.* (2000 ▶); Samadhiya & Halve (2001 ▶). For the synthesis of Schiff bases, see: Siddiqui *et al.* (2006 ▶); Iqbal *et al.* (2007 ▶). For applications of Schiff bases, see: Mookherjee *et al.* (1989 ▶); Kumar *et al.* (2009 ▶). For graph-set notation, see: Bernstein *et al.* (1995 ▶).
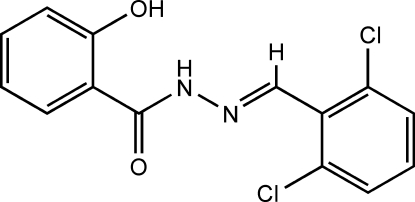


## Experimental

### 

#### Crystal data

C_14_H_10_Cl_2_N_2_O_2_
*M* *_r_* = 309.14
Monoclinic, 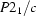

*a* = 7.5029 (6) Å
*b* = 23.8363 (13) Å
*c* = 8.0286 (7) Å
β = 109.860 (6)°
*V* = 1350.45 (18) Å^3^
*Z* = 4
Mo *K*α radiation
μ = 0.48 mm^−1^
*T* = 180 K
0.34 × 0.26 × 0.18 mm

#### Data collection

Stoe IPDS-2T diffractometer
Absorption correction: multi-scan (*SADABS*; Bruker, 2007 ▶) *T*_min_ = 0.856, *T*_max_ = 0.921
21101 measured reflections
2958 independent reflections
2455 reflections with *I* > 2σ(*I*)
*R*_int_ = 0.041

#### Refinement

*R*[*F* ^2^ > 2σ(*F* ^2^)] = 0.029
*wR*(*F* ^2^) = 0.074
*S* = 1.03
2958 reflections
191 parameters
H atoms treated by a mixture of independent and constrained refinement
Δρ_max_ = 0.24 e Å^−3^
Δρ_min_ = −0.39 e Å^−3^

### 

Data collection: *X-AREA* (Stoe & Cie, 1999 ▶); cell refinement: *X-AREA*; data reduction: *X-AREA*; program(s) used to solve structure: *SHELXS97* (Sheldrick, 2008 ▶); program(s) used to refine structure: *SHELXL97* (Sheldrick, 2008 ▶); molecular graphics: *ORTEP-3 for Windows* (Farrugia, 1997 ▶) and *PLATON* (Spek, 2009 ▶); software used to prepare material for publication: *WinGX* (Farrugia, 1999 ▶) and *PLATON*.

## Supplementary Material

Crystal structure: contains datablocks I, global. DOI: 10.1107/S1600536810035385/im2223sup1.cif

Structure factors: contains datablocks I. DOI: 10.1107/S1600536810035385/im2223Isup2.hkl

Additional supplementary materials:  crystallographic information; 3D view; checkCIF report

## Figures and Tables

**Table 1:** Hydrogen-bond geometry (Å, °)

*D*—H⋯*A*	*D*—H	H⋯*A*	*D*⋯*A*	*D*—H⋯*A*
N1—H1⋯N2^i^	0.85 (2)	2.38 (2)	3.165 (2)	153 (2)
N1—H1⋯O1^i^	0.85 (2)	2.45 (2)	3.158 (2)	140 (1)
O2—H2*A*⋯O1	0.90 (2)	1.78 (2)	2.608 (2)	153 (2)

Symmetry code: (i) 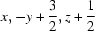.

**Fig. 1.**
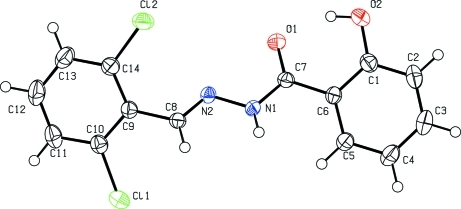
Molecular structure of the title compound with thermal ellipsoids drawn at the 50% probability level.

**Fig. 2.**
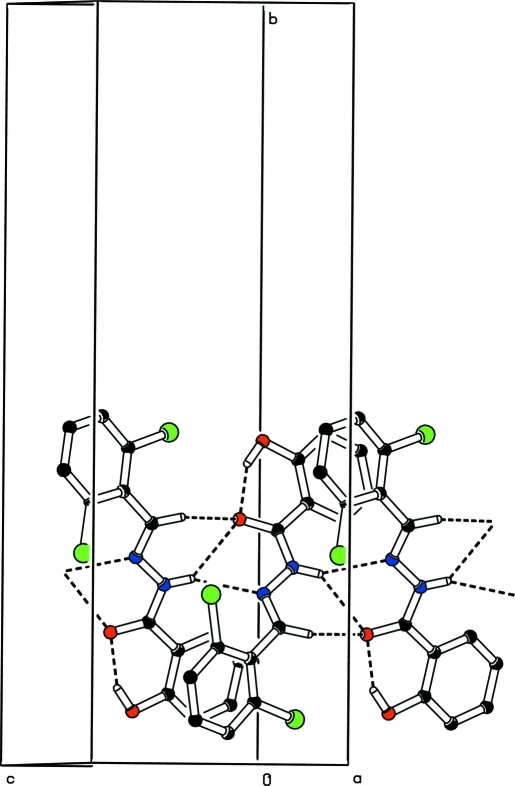
Packing diagram of the crystal structure showing hydrogen bonding as dashed lines.

## supplementary crystallographic information

### Comment

Schiff base reaction products of alkyl anthranilates and their derivatives have been employed in augmenting the aroma or taste of consumable materials including perfume compositions, colognes, perfumed articles, foodstuffs, chewing gums and beverages (Mookherjee *et al.*, 1989). Related compounds have also shown to exhibit biological activities such as antibacterial, antimicrobial (El-Masry *et al.*, 2000), and were investigated as herbicides (Samadhiya *et al.*, 2001). Further, Schiff bases have also been employed as ligands for complexation of metal ions (Kumar *et al.*, 2009). With this perspective of widespread applications of Schiff bases we embarked on the synthesis, characterization and biological evaluation of this class of compounds (Siddiqui *et al.*, 2006; Iqbal *et al.*, 2007). Herein, we report the synthesis and crystal structure of the title compound.

The title compound is presented in Fig.1. The two aromatic ring systems in the hydrazide are inclined at an angle of 17.39 (0.04) ° with respect to each other. The structure possesses classical inter and intra molecular hydrogen bonding. The intramolecular O–H···O type hydrogen bonding forms six membered ring motif *R*(6)_1_^1^ (Bernstein *et al.*, 1995) which inclines at an angle of 9.73 (0.14) ° with respect to aromatic C1–C6. The intermolecular C–H···O and N—H···N type of hydrogen bonding forms nine membered ring motif *R*(9)_2_^2^ (Bernstein *et al.*, 1995) where N–H···O type of hydrogen bonding interveins to form a six and a five membered ring system *R*(6)_2_^1^ and *R*(5)_1_^2^(Bernstein *et al.*, 1995), respectively (Fig. 2, table 1).

### Experimental

A mixture of 2-hydroxy-benzoic acid hydrazide (1.5 g, 10.0 mmol) and 2,6-dichlorobenzaldehyde (1.7 g, 10.0 mmol) in absolute ethanol (20 ml) was heated to reflux (2 hrs.), cooled to room temperature and filtered. The off-white precipitates were washed with the same solvent and dried at room temperature to yield 2.8 g of off-white, needle-like crystals of the title compound (9.1 mmol, 90.6%). Suitable crystals were grown from a solution of CH_3_OH by slow evaporation at room temperature.

### Refinement

All aromatic H-atoms were positioned geometrically with C—H = 0.95 Å and refined using riding model with *U*_iso_(H) = 1.2 *U*_eq_(C), while the imine hydrogen was located in difference map and was refined with C—H = 0.95 (2) Å and *U*_iso_(H) = 1.2 *U*_eq_(C8). N–H and O–H H atoms also were located in difference map and were refined with N—H = 0.86 (2) Å and O—H = 0.89 (2) Å and *U*_iso_(H) = 1.2 *U*_eq_(N) and *U*_iso_(H) = 1.5 *U*_eq_(O), respectively.

### Crystal data

**Table:**

C_14_H_10_Cl_2_N_2_O_2_	*F*(000) = 632
*M**_r_* = 309.14	*D*_x_ = 1.520 Mg m^−^^3^
Monoclinic, *P*2_1_/*c*	Mo *K*α radiation, λ = 0.71073 Å
Hall symbol: -P 2ybc	Cell parameters from 19179 reflections
*a* = 7.5029 (6) Å	θ = 1.7–29.5°
*b* = 23.8363 (13) Å	µ = 0.48 mm^−^^1^
*c* = 8.0286 (7) Å	*T* = 180 K
β = 109.860 (6)°	Needles, white
*V* = 1350.45 (18) Å^3^	0.34 × 0.26 × 0.18 mm
*Z* = 4	

### Data collection

**Table:**

Stoe IPDS-2T diffractometer	2958 independent reflections
Radiation source: fine-focus sealed tube	2455 reflections with *I* > 2σ(*I*)
graphite	*R*_int_ = 0.041
ω scans	θ_max_ = 27.0°, θ_min_ = 1.7°
Absorption correction: multi-scan (*SADABS*; Bruker, 2007)	*h* = −9→9
*T*_min_ = 0.856, *T*_max_ = 0.921	*k* = −30→30
21101 measured reflections	*l* = −10→10

### Refinement

**Table:**

Refinement on *F*^2^	Secondary atom site location: difference Fourier map
Least-squares matrix: full	Hydrogen site location: inferred from neighbouring sites
*R*[*F*^2^ > 2σ(*F*^2^)] = 0.029	H atoms treated by a mixture of independent and constrained refinement
*wR*(*F*^2^) = 0.074	*w* = 1/[σ^2^(*F*_o_^2^) + (0.0408*P*)^2^ + 0.2354*P*] where *P* = (*F*_o_^2^ + 2*F*_c_^2^)/3
*S* = 1.03	(Δ/σ)_max_ = 0.001
2958 reflections	Δρ_max_ = 0.24 e Å^−^^3^
191 parameters	Δρ_min_ = −0.39 e Å^−^^3^
0 restraints	Extinction correction: *SHELXL97* (Sheldrick, 2008), Fc^*^=kFc[1+0.001xFc^2^λ^3^/sin(2θ)]^-1/4^
Primary atom site location: structure-invariant direct methods	Extinction coefficient: 0.0085 (19)

### Special details

**Table:**

Geometry. All s.u.'s (except the s.u. in the dihedral angle between two l.s. planes) are estimated using the full covariance matrix. The cell s.u.'s are taken into account individually in the estimation of s.u.'s in distances, angles and torsion angles; correlations between s.u.'s in cell parameters are only used when they are defined by crystal symmetry. An approximate (isotropic) treatment of cell s.u.'s is used for estimating s.u.'s involving l.s. planes.
Refinement. Refinement of *F*^2^ against ALL reflections. The weighted *R*-factor *wR* and goodness of fit *S* are based on *F*^2^, conventional *R*-factors *R* are based on *F*, with *F* set to zero for negative *F*^2^. The threshold expression of *F*^2^ > 2σ(*F*^2^) is used only for calculating *R*-factors(gt) *etc*. and is not relevant to the choice of reflections for refinement. *R*-factors based on *F*^2^ are statistically about twice as large as those based on *F*, and *R*- factors based on ALL data will be even larger.

### Fractional atomic coordinates and isotropic or equivalent isotropic displacement parameters (Å^2^)

**Table:**

	*x*	*y*	*z*	*U*_iso_*/*U*_eq_	
Cl1	0.66364 (6)	0.562338 (15)	0.52580 (5)	0.03982 (12)	
Cl2	0.28455 (5)	0.723630 (17)	0.06158 (5)	0.03924 (12)	
O1	0.77275 (17)	0.82495 (4)	0.34242 (14)	0.0369 (3)	
O2	0.84369 (18)	0.92793 (5)	0.45517 (16)	0.0431 (3)	
H2A	0.809 (3)	0.8981 (10)	0.383 (3)	0.065*	
N1	0.72285 (16)	0.76179 (5)	0.53258 (15)	0.0239 (2)	
H1	0.720 (2)	0.7541 (7)	0.636 (2)	0.029*	
N2	0.63961 (16)	0.72631 (5)	0.39193 (15)	0.0233 (2)	
C1	0.9163 (2)	0.90454 (6)	0.6187 (2)	0.0308 (3)	
C2	1.0154 (2)	0.93943 (7)	0.7591 (2)	0.0383 (4)	
H2	1.0262	0.9784	0.7396	0.046*	
C3	1.0976 (2)	0.91717 (7)	0.9261 (2)	0.0409 (4)	
H3	1.1648	0.9411	1.0215	0.049*	
C4	1.0837 (2)	0.86024 (7)	0.9572 (2)	0.0374 (4)	
H4	1.1431	0.8452	1.0723	0.045*	
C5	0.9828 (2)	0.82576 (6)	0.81912 (18)	0.0281 (3)	
H5	0.9726	0.7869	0.8404	0.034*	
C6	0.89542 (19)	0.84719 (6)	0.64844 (18)	0.0246 (3)	
C7	0.79269 (19)	0.81097 (6)	0.49710 (18)	0.0246 (3)	
C8	0.56322 (19)	0.68201 (6)	0.42645 (18)	0.0242 (3)	
H8	0.562 (2)	0.6739 (7)	0.542 (2)	0.029*	
C9	0.47973 (19)	0.64095 (6)	0.28383 (17)	0.0252 (3)	
C10	0.5196 (2)	0.58369 (6)	0.31608 (19)	0.0286 (3)	
C11	0.4494 (2)	0.54288 (7)	0.1881 (2)	0.0379 (4)	
H11	0.4807	0.5045	0.2145	0.045*	
C12	0.3331 (3)	0.55883 (8)	0.0214 (2)	0.0439 (4)	
H12	0.2849	0.5313	−0.0681	0.053*	
C13	0.2862 (2)	0.61464 (8)	−0.0162 (2)	0.0406 (4)	
H13	0.2048	0.6254	−0.1307	0.049*	
C14	0.3588 (2)	0.65488 (6)	0.11426 (19)	0.0297 (3)	

### Atomic displacement parameters (Å^2^)

**Table:**

	*U*^11^	*U*^22^	*U*^33^	*U*^12^	*U*^13^	*U*^23^
Cl1	0.0478 (2)	0.02692 (19)	0.0405 (2)	0.00367 (16)	0.00954 (17)	0.00623 (15)
Cl2	0.03272 (19)	0.0378 (2)	0.0399 (2)	−0.00243 (15)	0.00277 (15)	0.01388 (16)
O1	0.0530 (7)	0.0320 (6)	0.0225 (5)	−0.0099 (5)	0.0088 (5)	0.0033 (4)
O2	0.0526 (7)	0.0255 (5)	0.0402 (7)	−0.0026 (5)	0.0013 (5)	0.0067 (5)
N1	0.0302 (6)	0.0239 (6)	0.0174 (5)	−0.0030 (4)	0.0079 (5)	−0.0014 (4)
N2	0.0253 (5)	0.0229 (5)	0.0214 (5)	0.0000 (4)	0.0077 (4)	−0.0026 (4)
C1	0.0309 (7)	0.0255 (7)	0.0350 (8)	0.0002 (5)	0.0097 (6)	0.0000 (6)
C2	0.0373 (8)	0.0269 (7)	0.0494 (9)	−0.0047 (6)	0.0130 (7)	−0.0102 (7)
C3	0.0394 (8)	0.0431 (9)	0.0382 (9)	−0.0097 (7)	0.0104 (7)	−0.0166 (7)
C4	0.0375 (8)	0.0470 (9)	0.0261 (7)	−0.0070 (7)	0.0088 (6)	−0.0059 (6)
C5	0.0273 (7)	0.0315 (7)	0.0257 (7)	−0.0033 (5)	0.0092 (6)	−0.0009 (5)
C6	0.0237 (6)	0.0254 (7)	0.0249 (7)	−0.0003 (5)	0.0084 (5)	−0.0026 (5)
C7	0.0269 (7)	0.0229 (6)	0.0232 (7)	0.0022 (5)	0.0076 (5)	0.0011 (5)
C8	0.0274 (7)	0.0229 (6)	0.0225 (7)	0.0012 (5)	0.0087 (5)	0.0011 (5)
C9	0.0272 (6)	0.0257 (7)	0.0250 (7)	−0.0032 (5)	0.0118 (5)	−0.0010 (5)
C10	0.0310 (7)	0.0272 (7)	0.0305 (7)	−0.0025 (6)	0.0142 (6)	−0.0012 (5)
C11	0.0469 (9)	0.0279 (7)	0.0450 (9)	−0.0067 (6)	0.0238 (8)	−0.0096 (7)
C12	0.0539 (10)	0.0437 (10)	0.0386 (9)	−0.0164 (8)	0.0214 (8)	−0.0176 (7)
C13	0.0431 (9)	0.0520 (10)	0.0255 (7)	−0.0143 (8)	0.0102 (7)	−0.0048 (7)
C14	0.0299 (7)	0.0324 (7)	0.0279 (7)	−0.0058 (6)	0.0114 (6)	0.0022 (6)

### Geometric parameters (Å, °)

**Table:**

Cl1—C10	1.7395 (15)	C4—H4	0.9500
Cl2—C14	1.7364 (16)	C5—C6	1.399 (2)
O1—C7	1.2448 (17)	C5—H5	0.9500
O2—C1	1.3582 (19)	C6—C7	1.4763 (18)
O2—H2A	0.90 (2)	C8—C9	1.4745 (19)
N1—C7	1.3533 (18)	C8—H8	0.954 (17)
N1—N2	1.3788 (15)	C9—C14	1.396 (2)
N1—H1	0.854 (18)	C9—C10	1.402 (2)
N2—C8	1.2761 (18)	C10—C11	1.383 (2)
C1—C2	1.395 (2)	C11—C12	1.379 (3)
C1—C6	1.406 (2)	C11—H11	0.9500
C2—C3	1.378 (2)	C12—C13	1.383 (3)
C2—H2	0.9500	C12—H12	0.9500
C3—C4	1.390 (3)	C13—C14	1.388 (2)
C3—H3	0.9500	C13—H13	0.9500
C4—C5	1.381 (2)		
			
C1—O2—H2A	103.3 (15)	O1—C7—C6	121.12 (12)
C7—N1—N2	117.32 (11)	N1—C7—C6	117.68 (12)
C7—N1—H1	121.9 (11)	N2—C8—C9	118.97 (12)
N2—N1—H1	120.5 (11)	N2—C8—H8	122.3 (10)
C8—N2—N1	116.20 (11)	C9—C8—H8	118.7 (10)
O2—C1—C2	117.72 (14)	C14—C9—C10	116.02 (13)
O2—C1—C6	122.16 (13)	C14—C9—C8	124.31 (13)
C2—C1—C6	120.12 (14)	C10—C9—C8	119.67 (12)
C3—C2—C1	119.77 (15)	C11—C10—C9	122.96 (15)
C3—C2—H2	120.1	C11—C10—Cl1	117.91 (12)
C1—C2—H2	120.1	C9—C10—Cl1	119.14 (11)
C2—C3—C4	120.96 (15)	C12—C11—C10	118.86 (15)
C2—C3—H3	119.5	C12—C11—H11	120.6
C4—C3—H3	119.5	C10—C11—H11	120.6
C5—C4—C3	119.43 (15)	C11—C12—C13	120.47 (15)
C5—C4—H4	120.3	C11—C12—H12	119.8
C3—C4—H4	120.3	C13—C12—H12	119.8
C4—C5—C6	121.02 (14)	C12—C13—C14	119.64 (16)
C4—C5—H5	119.5	C12—C13—H13	120.2
C6—C5—H5	119.5	C14—C13—H13	120.2
C5—C6—C1	118.64 (13)	C13—C14—C9	122.02 (15)
C5—C6—C7	122.17 (12)	C13—C14—Cl2	117.22 (12)
C1—C6—C7	119.07 (12)	C9—C14—Cl2	120.70 (11)
O1—C7—N1	121.20 (12)		
			
C7—N1—N2—C8	−175.33 (12)	N1—N2—C8—C9	−177.11 (11)
O2—C1—C2—C3	−177.58 (15)	N2—C8—C9—C14	−47.26 (19)
C6—C1—C2—C3	1.9 (2)	N2—C8—C9—C10	133.40 (14)
C1—C2—C3—C4	0.1 (2)	C14—C9—C10—C11	1.8 (2)
C2—C3—C4—C5	−1.3 (2)	C8—C9—C10—C11	−178.78 (13)
C3—C4—C5—C6	0.4 (2)	C14—C9—C10—Cl1	−178.18 (10)
C4—C5—C6—C1	1.6 (2)	C8—C9—C10—Cl1	1.21 (18)
C4—C5—C6—C7	177.71 (13)	C9—C10—C11—C12	−0.7 (2)
O2—C1—C6—C5	176.71 (13)	Cl1—C10—C11—C12	179.27 (12)
C2—C1—C6—C5	−2.8 (2)	C10—C11—C12—C13	−0.6 (2)
O2—C1—C6—C7	0.5 (2)	C11—C12—C13—C14	0.8 (2)
C2—C1—C6—C7	−178.98 (13)	C12—C13—C14—C9	0.4 (2)
N2—N1—C7—O1	4.02 (19)	C12—C13—C14—Cl2	−176.77 (13)
N2—N1—C7—C6	−175.26 (11)	C10—C9—C14—C13	−1.6 (2)
C5—C6—C7—O1	−155.05 (14)	C8—C9—C14—C13	179.01 (14)
C1—C6—C7—O1	21.0 (2)	C10—C9—C14—Cl2	175.41 (10)
C5—C6—C7—N1	24.23 (19)	C8—C9—C14—Cl2	−3.95 (19)
C1—C6—C7—N1	−159.71 (13)		

### Hydrogen-bond geometry (Å, °)

**Table:**

*D*—H···*A*	*D*—H	H···*A*	*D*···*A*	*D*—H···*A*
N1—H1···N2^i^	0.85 (2)	2.38 (2)	3.165 (2)	153 (2)
N1—H1···O1^i^	0.85 (2)	2.45 (2)	3.158 (2)	140 (1)
O2—H2A···O1	0.90 (2)	1.78 (2)	2.608 (2)	153 (2)

Symmetry codes: (i) *x*, −*y*+3/2, *z*+1/2.
